# *Fusarium Solani* Infection Following Burn Injury: A Case Report

**DOI:** 10.29252/wjps.8.3.406

**Published:** 2019-09

**Authors:** Naeem Goussous, Anas Abdullah, Stephen M. Milner

**Affiliations:** Johns Hopkins Burn Center, Michael D. Hendrix Burn Research Center, Johns Hopkins University School of Medicine, Baltimore, USA

**Keywords:** *Fusarium solani*, Fungus, Mold; Infection, Burn

## Abstract

Fungal infections are becoming increasingly recognized among burn patients. Infection with Fusarium, a filamentous mold, is rarely encountered and mainly seen in immunocompromised patients. High mortality and morbidity were reported with these virulent infections. We present a rare case of refractory septic shock from upper extremity fungal infection with *Fusarium solani* in a burn patient. Multiple operative debridements and below elbow amputation caused resolution of septic shock. Closure was achieved with a split thickness skin graft. Aggressive approach should be adopted in managing burn patients with Fusarium infection. Serial debridements and extremity amputation should be considered in attempts to improve survival.

## INTRODUCTION

Fusarium is a saprophytic filamentous mold found ubiquitously in the soil and water. Infection with this organism has been reported in patients with suppressed immunity in conditions including: leukemia, lymphoma, aplastic anemia, and those on chemotherapy. *Fusarium solani* is the most frequently isolated species, where high mortality rates have been associated ranging from 40-50%. Burned skin acts as a portal for entry of fungi and the impaired immune status facilitates deep invasion.^1^

The practice of early excision of burn wounds and the application of topical antibacterial agents have led to decreased incidence of bacterial wound infections. Despite these advances, the incidence of fungal infections continues to be unchanged.^2^^-^^5^ *Candida* species have been the most commonly cultured organisms; although non-candidal infections are associated with a higher risk of tissue invasiveness and mortality.^6^ A recent study from Australia demonstrated a 25% mortality rate in burn patients with non-candidal mold infection despite aggressive therapy with systemic antifungal agents and surgical debridement.^7^ Reports of infections with *Fusarium* are sparse in the literature. We present a case of cutaneous fusarial infection in a patient who sustained severe burn injury.

## CASE REPORT

A 55-year-old male presented initially to an outside facility after being found unconscious in a house fire. He sustained a 35% total body surface area (TBSA) full thickness burn to his back, upper chest and bilateral upper extremities along with an inhalational injury. His carboxy hemoglobin at presentation was 28.9%. He was intubated, resuscitated and treated with hyperbaric oxygen. After 48 hours post-injury, the patient was transferred to our facility. Examination revealed bilateral swollen upper extremities with compartment syndrome from circumferential deep burns. 

Escharotomies were performed promptly at the bedside. The patient developed a fever with leukocytosis. *H. infleunzae* and *S. aureus* were isolated from sputum and blood, respectively. He was started on piperacillin/tazobactam and vancomycin. He then underwent staged excision of his burn wounds. Twelve days after admission, he developed septic shock and acute kidney injury requiring hemodialysis. Examination of the wounds in the left upper extremity revealed friable tissues with blackish discoloration of the underlying deep tissues and muscles with a yellowish discharge and a bad odor. 

**Fig. 1:**
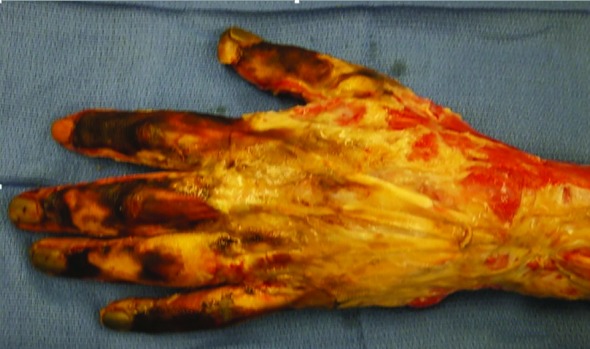
Intra-operative image of the left hand and distal forearm, extensor aspect

**Fig. 2:**
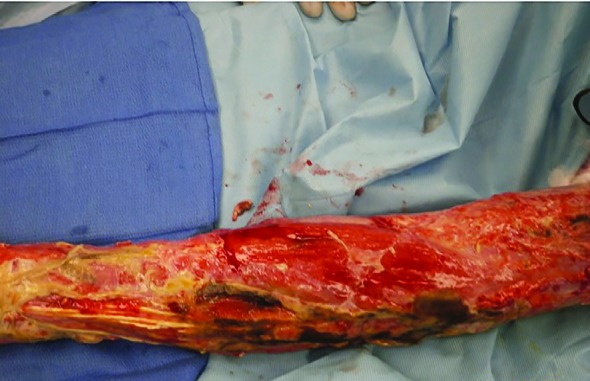
Intra-operative picture of the left forearm, dorsal aspect, showing necrosis around the extensor tendons

**Fig. 3:**
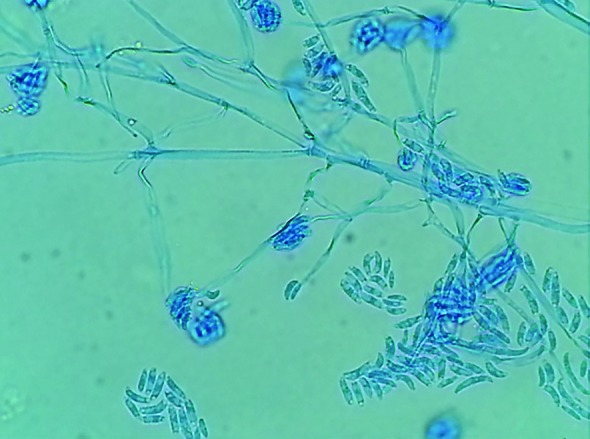
*F. solani* under light microscopy with a lactophenol cotton blue stain

Following further operative debridement and exploration the interossei muscles and the extensors of the forearm were necrotic (Figures 1 and 2). Initial results of the tissue cultures showed an unspecified mold, and the patient was treated with voriconazole. The mold was subsequently identified as *F. solani *(Figure 3). Despite the initiation of antifungal therapy and aggressive operative debridement, the patient’s condition continued to worsen; his white cell count continued to rise and peaked at 30,000 and the vasopressor requirement continued to escalate.

**Fig. 4:**
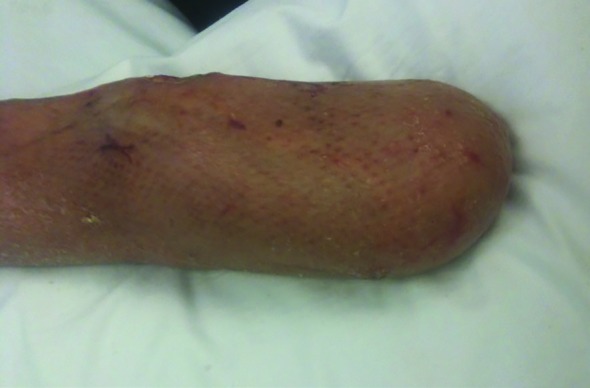
Appearance of the left upper stump at discharge

After further examination, the forearm deemed to be unsalvageable and due to concerns of worsening septic shock with the risk of hematogenous dissemination of the mold, he underwent left below elbow amputation and placement of a split thickness skin graft. Soon after the surgery, his clinical status showed remarkable improvement enabling weaning the vasopressor support and resolution of his sepsis. The rest of his hospital course was uneventful, the left upper extremity stump showed successful recovery and engraftment (Figure 4). He was discharged to an inpatient rehabilitation center for further physical therapy.

## DISCUSSION

*Fusarium* infections are mainly encountered in immunocompromised patients. These infections are limited to a few case reports and small case series with an incidence of 0.06-1.4% of fungal infected burn wounds.^7^^,^^8^ All cases shared a rapidly progressive deterioration in the clinical status with aggressive deep tissue invasion, progression of necrosis and distant dissemination. The mortality was almost universal.^9^^-^^11^ Diagnosis of these infections begins by having a high clinical suspicion. Several risk factors have been identified that predispose to develop invasive fungal infections including increasing burned TBSA, length of hospital stay, polymicrobial infections and the presence of inhalational injury.^4^^,^^5^

Tissue biopsy should be obtained immediately for both tissue culture and histopathological examination. Vascular invasion with thrombosis and tissue necrosis has been observed with fusarial infections. On microscopic examination, there was a close similarity between *Aspergillus* and *Fusarium*. The identification of *F. solani* can be challenging and may take up to 5-7 days to speciate. The addition of PCR can aid in the diagnosis.^12^^,^^13^ Treatment of fusarial infections center on surgical debridement and initiation of antifungal therapy. Early and aggressive surgical excision of all necrotic tissues is paramount.^1^

The initiation of antifungal therapy should be prompt and started as soon as the fungus is identified on light microscopy and before awaiting further speciation. Amphotericin B or voriconazole as a monotherpay has been shown to have equivalent success rates. Barret *et al.* reported eradication of deep and superficial angioinvasive fungal infections with the use of topical nystatin at a concentration of 6,000,00 units/g in four pediatric burn patients.^14^ Invasive fungal infections are fortunately uncommon in our unit. The early excision of burns, restricted use of prophylactic antibiotics and administration of fluconazole in burns >40% may be responsible for our low rate.^15^

## CONFLICT OF INTEREST

The authors declare no conflict of interest.
